# The Role of Antioxidant Enzymes in Adaptive Responses to Sheath Blight Infestation under Different Fertilization Rates and Hill Densities

**DOI:** 10.1155/2014/502134

**Published:** 2014-07-17

**Authors:** Wei Wu, Xuejie Wan, Farooq Shah, Shah Fahad, Jianliang Huang

**Affiliations:** ^1^College of Agronomy, Northwest A&F University, Yangling, Shaanxi 712100, China; ^2^National Key Laboratory of Crop Genetic Improvement, MOA Key Laboratory of Crop Physiology, Ecology and Cultivation (The Middle Reaches of Yangtze River), College of Plant Science and Technology, Huazhong Agricultural University, Wuhan, Hubei 430070, China; ^3^Department of Agriculture, Abdul Wali Khan University, Mardan, Khyber Pakhtunkhwa, Pakistan

## Abstract

Sheath blight of rice, caused by *Rhizoctonia solani*, is one of the most devastating rice diseases worldwide. No rice cultivar has been found to be completely resistant to this fungus. Identifying antioxidant enzymes activities (activity of superoxide dismutase (SOD), peroxidase (POD), and catalase (CAT)) and malondialdehyde content (MDA) responding to sheath blight infestation is imperative to understand the defensive mechanism systems of rice. In the present study, two inoculation methods (toothpick and agar block method) were tested in double-season rice. Toothpick method had greater lesion length than agar block method in late season. A higher MDA content was found under toothpick method compared with agar block method, which led to greater POD and SOD activities. Dense planting caused higher lesion length resulting in a higher MDA content, which also subsequently stimulated higher POD and SOD activity. Sheath blight severity was significantly related to the activity of antioxidant enzyme during both seasons. The present study implies that rice plants possess a system of antioxidant protective enzymes which helps them in adaptation to sheath blight infection stresses. Several agronomic practices, such as rational use of fertilizers and optimum planting density, involved in regulating antioxidant protective enzyme systems can be regarded as promising strategy to suppress the sheath blight development.

## 1. Introduction

Sheath blight, caused by* Rhizoctonia solani Kühn* anastomosis group AG-1 IA, is a devastating disease in rice (*Oryza sativa *L.), especially in intensive production systems, such as a double rice-cropping system [1, 2]. Recently, the intensity of sheath blight in rice-growing regions has increased due to several agronomic practices, characterised by abundant nitrogenous fertiliser application, a high planting density, and the use of popular high-yielding hybrid cultivars [3, 4].

Control strategies for sheath blight have centered on the use of sprayed fungicides [5]. However, the use of fungicides is limited due to serious environmental problems, expense, and the potential risk of pathogen populations becoming less sensitive [6]. Plant resistance to sheath blight generally is considered to be polygenic, with a wide variation in susceptibility levels among rice cultivars [7]. The development of cultivars resistant to sheath blight can be a promising strategy for suppressing sheath blight. However, no rice cultivar has been found to be completely resistant to this fungus.

It is well-documented that agronomic practices (e.g., fertiliser management and hill density) play a pivotal role in suppression of sheath blight development [2]. Agronomic practices can significantly influence crop growth, altering tiller number, leaf area index, plant height, and shoot extension, all of which result in a variation in canopy structure. Dense canopy structure leads to a high contact frequency and thus facilitates the sheath blight epidemic in vertical phase [3, 8]. Furthermore, a number of environmental factors, such as humidity and temperature within the canopy, are altered concomitantly with the canopy architecture and canopy density, which can affect the spread of the rice sheath blight pathogen [1, 3]. Lastly, silicon fertiliser has the potential to complement inherent host resistance and to suppress sheath blight intensity [9]. These fertilisers can all contribute to variation in plant resistance to this disease [8, 10]. In addition, biochemical responses, including plant cellular antioxidant enzyme activities against disease stresses, are regarded as early warning indicators of injury, which are also related to resistance to sheath blight in rice [11]. However, the response of antioxidant enzyme activities in rice plants to disease stresses has been rarely discussed.

Antioxidant enzyme responses to environmental stress in rice have been primarily assessed for freezing, drought, salt, or other primary stresses [12]. It also showed that antioxidant enzyme activities in plants were stimulated to repair or to resist the damage caused by the collection of reactive oxygen species (ROS) due to environmental stresses [12]. Furthermore, antioxidant enzyme activities have been shown to contribute significantly to plant resistance to abiotic and biotic stresses. We hypothesise that severe sheath blight could also stimulate higher antioxidant enzyme activities in rice plants.

To facilitate detailed analysis of the response of antioxidant enzyme activities to different sheath blight resistance levels, different agronomic practices (including N rate, supplemental silicon, and hill density) and two sheath blight inoculation methods were employed under field conditions. Although many studies have shown the influence of N rate, silicon rate, hill density, and inoculation method on sheath blight development separately [3, 7, 9, 13], these studies provide limited information as to the combinatory treatment effects on sheath blight severity and their relationship with antioxidant enzyme activities in high-yielding hybrids. In addition, the present study assesses whether antioxidant enzyme activities can be regarded as the correct parameters to assess when evaluating sheath blight resistance levels. Thus, the objectives of this study were to (i) determine the interactive effects between fertilisation rate and hill density on plant resistance to disease under two inoculation methods and to (ii) identify whether antioxidant enzyme activities (SOD, POD, and CAT) and MDA content respond to different disease resistance levels under various sheath blight inoculation methods, fertilisation rates, and hill densities.

## 2. Materials and Methods

### 2.1. Experimental Design and Cultural Practices

Field experiments were conducted over a double season in 2009 at Dajin, Wuxue, Hubei, China (29°59′N 115°36′E). The soil properties at the experimental site are described elsewhere [13].

The experiment was begun in the early season (April to July) and repeated in the late season (July to October) in the same field. During the two seasons, treatments were arranged in a split-split plot design with fertilisation rate as main plot, hill density as the subplot treatment, and inoculation methods as sub-subplot (microplot) treatments, respectively. The individual subplot size was 5 × 6 m. For microplots, two groups of 24 hills (4 × 6) in the centre of each subplot were selected for inoculation with the two methods, at the PI stage. Microplots in each subplot were separated from each other by an eight-hill-wide border. Four field experiments had four replications.

The main plot treatments were three fertiliser rates: zero N control, high N rate (N = 180 and 195 kg N ha^−1^ in early and late season, resp.), and high N rate with silicon supplementation (silicon = 76 kg Si ha^−1^). Subplot treatments were three hill densities: 37.5, 28.1, and 14.1 hills m^–2^, representing hill spacings of 13.3 × 20 cm, 13.3 × 26.7 cm, and 26.7 × 26.7 cm, respectively.

Nitrogen in the form of urea was split-applied at the basal (BS), mid-tillering (MT), panicle initiation (PI), and flowering (FL) stages. N-splitting patterns for the early and late seasons were 35% (BS) + 20% (MT) + 30% (PI) + 15% (FL) and 41% (BS) + 24% (MT) + 35% (PI), respectively. Phosphorus at 40 kg P ha^−1^, potassium at 50 kg K ha^−1^, and zinc at 5 kg Zn ha^−1^ were applied as a basal application in all plots. The basal fertilisers were broadcast and incorporated one day before transplanting. Additional potassium at 50 kg K ha^−1^ was top-dressed at PI in all plots. No fungicide was applied to any plot in either season.

Two widely adapted high-yielding F_1_ hybrid commercial cultivars are Liangyou-287, which was used in the early season, and T-you207, which was used in the late season, respectively. Early-season rice was sown on March 24 and transplanted on April 30. Late-season rice was sown on June 19 and transplanted on July 28. One or two seedlings were transplanted per hill in each experiment. Each main plot was surrounded by a 35 cm wide ridge covered with plastic film. The plastic film was installed to a depth of 20 cm below soil surface three days before transplanting. Crop management followed the standard cultural practices for the region. Insect and pests were controlled using insecticides to avoid biomass and yield loss. Diseases other than sheath blight were not observed in both trials.

### 2.2. Pathogen Inoculation and Assessment

An* R. solani* anastomosis group AG-1A isolate (WH-1 strain), which was isolated from typical sheath blight lesions in a rice field at Huazhong Agricultural University, Wuhan, China [14], was used to inoculate plots in each trial. Two inoculation methods, a short toothpick method [15], and an agar block method [16] were tested during both seasons. Either autoclaved 10 mm long wooden toothpicks or a 10 mm diameter agar block was incubated with the WH-1 strain on potato dextrose agar (PDA) at 28°C for 4 days. For the short toothpick method, infected toothpicks were inserted into the third sheath, counting from the top of the rice plant. Colonised agar blocks were placed on the surface of the third sheath, which was subsequently covered by cling film to maintain high humidity. In each subplot during both seasons, two groups of 24 hills (4 × 6) in the centre of each subplot were selected as microplots for inoculation using one of the two methods, at the PI stage (32 days after transplanting). Three main shoots from each inoculated hill were selected for inoculation in the microplots. In both seasons, lesion length (cm) was measured at 7 days after inoculation, based on 12 inoculated shoots per plot from four adjacent hills. To minimise variability, all records were recorded by one person in each trial.

At the third and seventh days after inoculation, eight leaves at the third internode (counting from the top of the plant) were randomly selected for measurement of physiological traits (malondialdehyde (MDA) content, activities of peroxidase (POD), catalase (CAT), and superoxide dismutase (SOD)). Methods for extractions of enzymes, assays of enzymes activity (POD, CAT, and SOD), and determination of MDA content are described in Liu et al. [17] and briefly described as follows: approximately 0.15 g fresh leaf tissue was homogenised in a precooled mortar in 5 mL of a 50 mmol/L phosphate buffer (pH 7.8) solution. The homogenate was centrifuged at 11000 g for 15 min at 5°C. The supernatant was used to determine enzyme activities (SOD, CAT, and POD) and MDA content. For the POD assay, 3 mL reaction solution contained 1 mL of 0.3% H_2_O_2_, 0.95 mL of 0.2% guaiacol, 1 mL of 50 mmol/L phosphate buffer (pH 7.0), and 0.05 mL enzyme extract. One unit of enzyme activity was defined as the amount of the enzyme that resulted in a 1% absorbance increase in 60 s at 470 nm. For the CAT assay, 0.1 mL enzyme extract was added to a solution of 1 mL of 0.3% H_2_O_2_ and 1.9 mL of 50 mmol/L phosphate buffer (pH 7.0). The activity of CAT was measured by determining the rate change of H_2_O_2_ absorbance in 60 s at 240 nm. For the SOD assay, 3 mL reaction mixture contained 0.3 mL of each of the following: 750 *μ*mol/L nitroblue tetrazolium, 20 *μ*mol/L riboflavin, 130 mmol/L methionine, and 100 *μ*mol/L EDTA-Na_2_, 1.5 mL of 50 mmol/L phosphate buffer (pH 7.8), 0.25 mL of deionised water, and 0.05 mL of enzyme extract. One unit of enzyme activity was defined as the amount of the enzyme that resulted in 50% inhibition of the rate of nitroblue tetrazolium (NBT) reduction. For the MDA assay, a solution containing 2.5 mL of 20% (w/v) trichloroacetic acid, 0.5% (w/v) thiobarbituric acid, and 1.5 mL enzyme extract was used. After placement in a boiling water bath, quick cooling, and refrigeration, the homogenate was centrifuged at 5000 g for 10 min at 25°C. The absorbance of the supernatant was recorded at 532 nm and 600 nm, respectively. After subtracting the nonspecific absorbance (600 nm), the MDA content was determined by its molar extinction coefficient (155 mM^−1 ^cm^−1^), and the results were expressed as *μ*mol MDA g^−1^ FW.

### 2.3. Data Analysis

**Figure 1:**
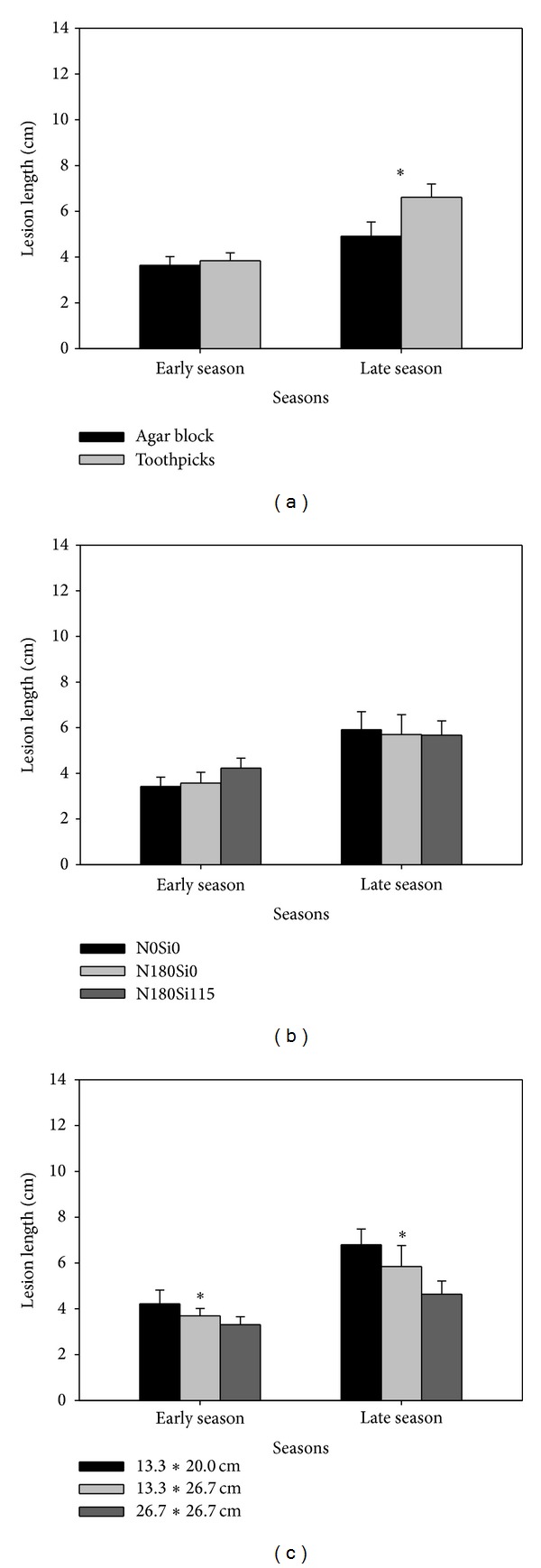
Lesion length as affected by (a) different inoculation methods, (b) fertilization rates, and (c) hill densities in double-season rice. Vertical bars indicate standard error. ∗ indicates significant difference between treatments according to LSD (0.05).

The data were subjected to an analysis of variance for each season [18]. In each season, the variance of lesion length among different treatments was analysed using a split-split-plot design with fertilisation rate as main plot, hill density as the subplot treatment, and inoculation methods as sub-subplots (microplot). Because the two- or three-way interactive effects among fertilisation rate, hill densities, and inoculation treatments on lesion length were not significant (data not shown), these traits shown in Figure 1 represent the mean data across the other two treatments. Data for antioxidant enzyme activities (POD, CAT, and SOD) and MDA content were averaged across the two seasons. Relationships of lesion length to POD, CAT, SOD, and MDA for both seasons were evaluated using correlation analyses [19]. The means among treatments were compared using the least significant difference test (LSD) at a probability level of 0.05.

## 3. Results

No significant interaction effects for lesion length were found among the different treatments for both seasons (data not shown). Lesion length was significantly increased for the toothpick method and was higher than that for the agar block method in the late season, although no significant increase was found in the early season (Figure 1(a)). Fertilisation rate had no significant effects on lesion length (Figure 1(b)). Increased hill density significantly increased lesion length in the two seasons (Figure 1(c)).

**Figure 2:**
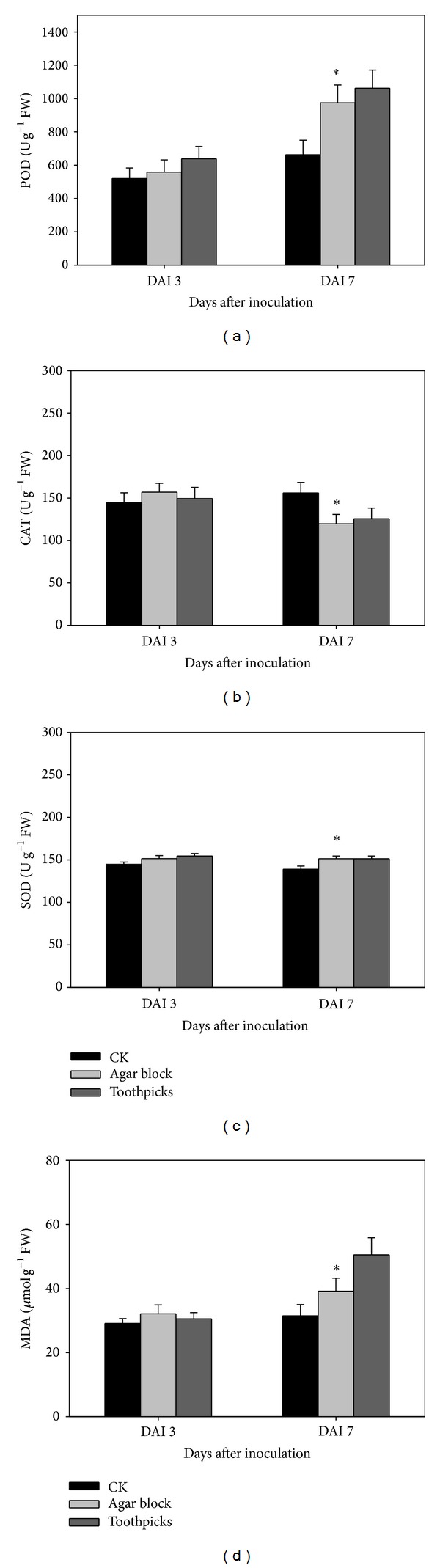
Effect of inoculation methods on antioxidant enzyme activities and MDA content 3 and 7 days after inoculation (DAI) in double-season rice. Vertical bars indicate standard error. ∗ indicates significant difference between treatments according to LSD (0.05).

**Figure 3:**
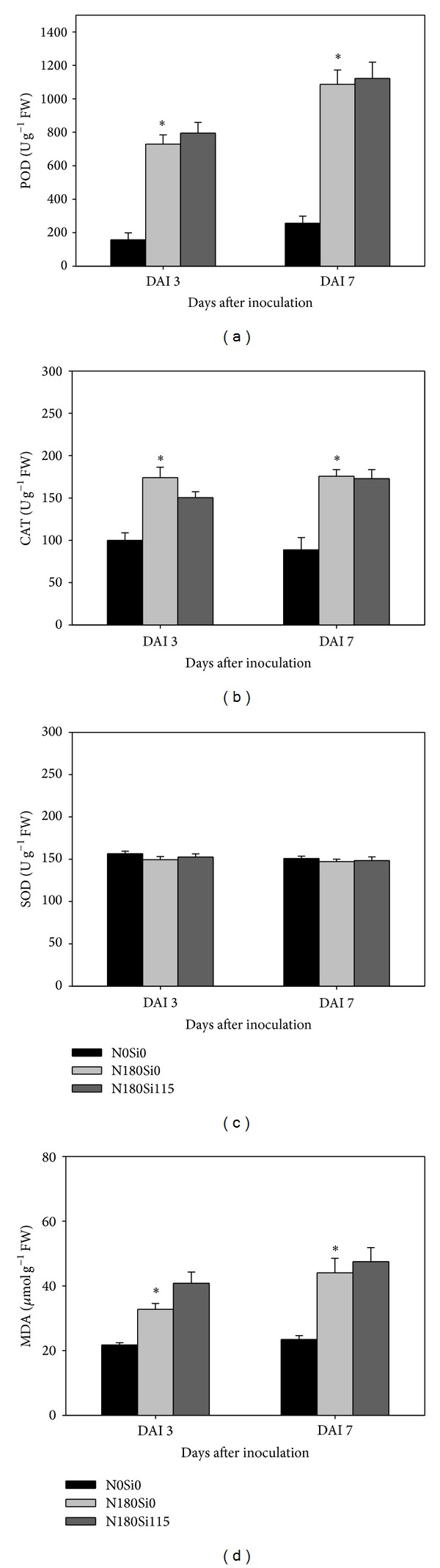
Effect of fertilization rates on antioxidant enzyme activities and MDA content 3 and 7 days after inoculation (DAI) in double-season rice. Vertical bars indicate standard error. ∗ indicates significant difference between treatments according to LSD (0.05).

**Figure 4:**
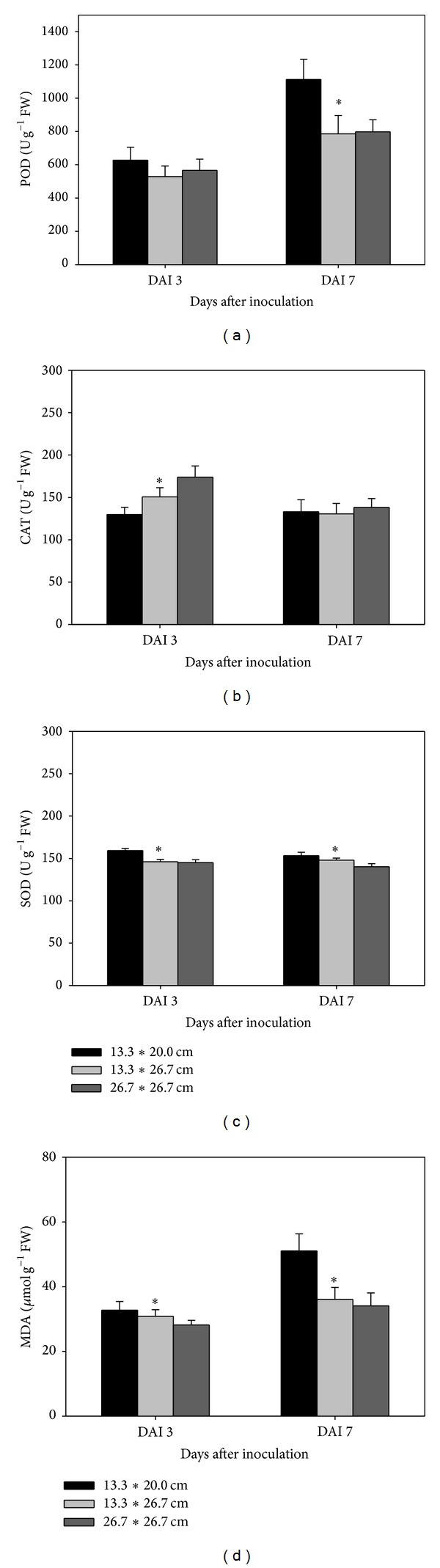
Effect hill densities on antioxidant enzyme activities and MDA content 3 and 7 days after inoculation (DAI) in double-season rice. Vertical bars indicate standard error. ∗ indicates significant difference between treatments according to LSD (0.05).

POD and MDA increased with time after inoculation, but CAT and SOD did not show a similar trend (Figures (2)–(4)). POD, SOD, and MDA were significantly increased for the toothpick method, compared with CK (no pathogen inoculation), followed by the agar block method at 7 days after inoculation rather than at 3 days after inoculation (Figure 2). However, CAT was significantly lower for the agar block and toothpick methods than for CK at 7 days after inoculation. N application or supplemental silicon significantly increased POD, CAT, and MDA at 3 and 7 days after inoculation. However, this was not the case for SOD (Figure 3). Increased hill density significantly increased POD, SOD, and MDA at 7 days after inoculation (Figure 4). Correlation analysis showed that lesion length had positive and significant relationships with POD, MDA, and SOD and had negative relationships with CAT at 7 days after inoculation during both seasons (Table 1). However, there are no significant correlations between lesion length and any antioxidant enzyme activities and MDA content at 3 days after inoculation.

## 4. Discussion

During both trials, sheath blight infection by two inoculation methods was nearly uniform and lesions were observed at 48 hours after inoculation regardless of fertilisation rate and hill density. Hundred percent infection rates were achieved due to higher humidity retention due to leaf sheath and cling film covering for the toothpick and agar block methods. Such findings are consistent with those of [15]. Furthermore, the toothpick method showed a significantly higher lesion length than the agar block method in the late season, which implies that toothpick method could be more efficient and faster than the agar block method.

The present study showed that increasing hill density increased lesion length significantly in both seasons. This may be explained by the higher canopy density, higher contact frequency, and higher moisture in the dense planting treatment than that in the sparse planting treatment (data not shown). This result was consistent with other reports that close spacing and high contact frequencies were favourable for sheath blight development in transplanted rice [13, 20].

Silicon fertiliser has the potential to increase the resistance of rice plants to sheath blight under greenhouse conditions [9]. In the present field studies, application of silicon fertiliser under high N rate did not show a significant negative effect on lesion length (Figure 1). The lack of response to silicon application most likely occurred for the following two reasons. Firstly, silicon concentration of the soil in this study was 84.0 mg kg^−1^, which was significantly higher than the critical level of 19.0 mg kg^−1^ [21]. Secondly, the soil used in this study was acidic such that silicon uptake from the soil by the rice plants was not constrained, as reported by [22].

These results also imply that, except for the fertilisation rate, the inoculation methods and hill density can lead to substantial variations in disease intensity. We hypothesised that different disease resistance levels can be related to various antioxidant enzymes activities. When suffering from stress such as infection with a disease, plant cells accumulate ROS, resulting in cell membrane lipid peroxidation and metabolic disorders, which lead to oxidative stress [23]. MDA content, the first product in membrane lipid peroxidation, is an index of the degree of injury to the plant cell [11]. In the present study, the MDA content in rice plants at 7 days after inoculation was significantly higher than that at 3 days after inoculation. This suggested that the degree of injury caused by the disease increased with a prolonged infection period in rice plants. The toothpick method, which showed a higher lesion length, was regarded as a more efficient inoculation method than the agar block method and also exhibited a higher MDA content at 7 days after inoculation. Similarly, dense planting resulted in a higher MDA content and a longer lesion length, compared to sparse planting.

A wide range of defensive mechanism systems, such as antioxidant protective enzyme systems, exists in higher plants to help them adapt to various biotic stresses [24, 25]. Among all the antioxidant enzymes, as the first defence mechanism against membrane lipid peroxidation caused by ROS, SOD can repair injured plant cells by catalysing the dismutation of O^2−^ to H_2_O_2_ and O_2_. Generally, high SOD activity leads to lower membrane lipid peroxidation [26, 27]. POD, which catalyses the reaction between H_2_O_2_ and ROOH to H_2_O and R-OH, ameliorates cell damage [17]. Meanwhile, CAT minimises plant injuries by inhibiting H_2_O_2_ production because when plants are exposed to a stressful condition, H_2_O_2_ is converted into HO, which results in lipid peroxidation, destruction of the electron transport chain in mitochondria and chloroplasts, and substantial damage to DNA and protein expression [23, 28, 29]. Therefore, the regulation of antioxidant enzyme systems in plants was shown to be associated with their ability to withstand biotic stresses caused by disease infection. In the present study, the POD activity was significantly higher in inoculation treatment (Figure 2(a)), N application treatment (Figure 3(a)), and sparse planting treatment (Figure 4(a)) at 7 days after inoculation. This indicated that, after inoculation, POD plays an important role in suppression of cell damage in rice plants, especially under high N and dense planting conditions. This conclusion was strongly supported by the close and significant relationships between lesion length and POD activity during both seasons (Table 1). Similarly, CAT was also induced when N and supplemental silicon were present. However, our study showed that SOD was not significantly stimulated by fertilisation or dense planting. Possibly, SOD activity was at a lower level in the rice plants because of the possible involvement of time factor in responding to disease stress. However, even at a low activity level, SOD also showed positive and significant relationships with disease severity in terms of lesion length (Table 1). A lack of a rapid response mechanism for increasing SOD activity to resist sheath blight may be another explanation for the question that why silicon did not exhibit a positive effect on the rice plants' resistance to sheath blight in the present study.

In summary, no interactive effects among fertilisation rate, hill density, and inoculation method on plant resistance to sheath blight were found. The toothpick method for inoculating rice plants caused higher lesion lengths than the agar block method. A higher MDA content was found for the toothpick method compared with the agar block method, which led to higher POD and SOD activities. Dense planting caused higher lesion length and resulted in a higher MDA content, which also subsequently stimulated higher POD and SOD activities. The present study implies that antioxidant protective enzyme systems indeed exist in rice plants to help them adapt to biotic stresses caused by sheath blight infection. Several agronomic practices involved in regulating antioxidant protective enzyme systems can be regarded as promising strategies for suppressing sheath blight development.

## Figures and Tables

**Table 1:** Relationships of lesion length (cm) with activity of peroxidase (POD), catalase (CAT), superoxide dismutase (SOD), and malondialdehyde (MDA) content in 2009 early and late seasons.

Seasons	Three days after inoculation				Seven days after inoculation			
	POD	CAT	SOD	MDA	POD	CAT	SOD	MDA
Early season	ns	ns	ns	ns	0.78∗	−0.86∗	0.77∗	0.57∗
Late season	ns	ns	ns	ns	0.76∗	−0.79∗	0.76∗	0.52∗

Levels of significance indicated: ns, not significant; ∗significant at *P* ≤ 0.05.
